# Effect of Five Different Stages of Ripening on Chemical Compounds in Medlar (*Mespilus germanica* L.)

**DOI:** 10.3390/molecules16010074

**Published:** 2010-12-28

**Authors:** Otakar Rop, Jiri Sochor, Tunde Jurikova, Ondrej Zitka, Helena Skutkova, Jiri Mlcek, Petr Salas, Boris Krska, Petr Babula, Vojtech Adam, Daniela Kramarova, Miroslava Beklova, Ivo Provaznik, Rene Kizek

**Affiliations:** 1 Department of Food Technology and Microbiology, Faculty of Technology, Tomas Bata University in Zlin, Namesti T. G. Masaryka 275, CZ-762 72 Zlin, Czech Republic; 2 Department of Breeding and Propagation of Horticultural Plants, Faculty of Horticulture, Mendel University in Brno, Valticka 337, CZ-691 44 Lednice, Czech Republic; 3 Department of Natural Drugs, Faculty of Pharmacy, University of Veterinary and Pharmaceutical Sciences Brno, Palackeho 1-3, CZ-612 42 Brno, Czech Republic; 4 Department of Natural and Informatics Sciences, Faculty of Central European Studies, Constantine the Philosopher University in Nitra, Drazovska 4, SK-949 74 Nitra, Slovak Republic; 5 Department of Chemistry and Biochemistry, Faculty of Agronomy, Mendel University in Brno, Zemedelska 1, CZ-613 00 Brno, Czech Republic; 6 Department of Biomedical Engineering, Faculty of Electrical Engineering and Communication, Brno University of Technology, Kolejni 4, CZ-612 00 Brno, Czech Republic; 7 Department of Fruit Growing, Faculty of Horticulture, Mendel University in Brno, Valticka 337, CZ-691 44 Lednice, Czech Republic; 8 Department of Food, Biochemistry and Analysis, Faculty of Technology, Tomas Bata University in Zlin, Namesti T. G. Masaryka 275, 762 72 Zlin, Czech Republic; 9 Department of Veterinary Ecology and Environmental Protection, Faculty of Veterinary Hygiene and Ecology, University of Veterinary and Pharmaceutical Sciences, Palackeho 1-3, CZ-612 42 Brno, Czech Republic

**Keywords:** medlar, minerals, ascorbic acid, phenolics, antioxidant activity, spectrometry, high performance liquid chromatography, nutrition

## Abstract

The study of changes of nutritional value of fruit during the ripening process can help estimate the optimal date for fruit harvesting to achieve the best quality for direct consumption and further utilization. The aim of this study was to monitor the changes of chemical composition of medlar fruit (*Mespilus germanica* L.) measured at five various ripening stages including 134, 144, 154, 164 and 174 days after full bloom (DAFB). Fruits were analyzed and ascorbic acid (AA) and total phenolic compound content with respect to the total antioxidant activity were determined. In addition, selected micronutrients and macronutrients were monitored. The results of our experiments demonstrate that ascorbic acid, total phenolic compound content and total antioxidant activity decreased significantly with increasing time of ripeness. The decreasing tendency in potassium, calcium and magnesium contents during the ripening stages was also determined. During the ripening period, the content of all micronutrients as well as phosphorus and sodium was balanced, with no statistically significant differences between the monitored ripening stages, which can be considered as a positive fact with respect to ideal consumption quality of fruit.

## 1. Introduction

Over thousands of years, fruit-producing rosaceous plants native to temperate latitudes of the Northern Hemisphere have played an important cultural role. The valuable fruits of this plant family have formed an integral part of the human diet. The family *Rosaceae*, comprised of over 100 genera and 3,000 species has recently been the third most economically important plant family in temperate regions [1]. The importance of edible rosaceous crops derives from both their nutritional and sensory qualities, which provide outstanding contributions to the dietary practice of consumers and overall human health [2,3,4,5,6]. Moreover, edible fruits of this family are extremely rich in compounds with strong antioxidant activities, such as L-ascorbic acid [7], phenolics including tannins [8], flavonoids, and other phytochemicals beneficial for health [9]. Despite the growing interest in less common fruit species as a source of new valuable compounds and their pharmacological properties, there are only minimal systematic efforts to estimate the value of less common or underutilized crops for this purpose. However, a wide range of dietary applications of phytochemicals has been found in commonly cultivated fruits of the *Rosaceae* family [10,11]. In addition, biochemical profiles of several fruits, such as *Amelanchier* or *Crataegus* have been estimated to provide new opportunities for both dietary and therapeutic purposes, such as the process of drug development [12]. Medlar (*Mespilus germanica* L.) belonging to *Amelanchier*-*Crataegus* sistergroup [13,14,15] is a spiny shrub, a member of the *Rosaceae* family which has been cultivated for many years in many countries of Europe and Asia for its edible fruits and ornamental qualities [16]. The medlar is also used in folk medicine, especially by the people of Southeastern Europe, Turkey and Iran, primarily for the treatment of constipation, as a diuretic, or to rid the kidney and bladder of stones. Medlar pulp or syrup is a popular remedy against enteritis [17]. The most common use of medlar fruits is however for raw consumption. Medlar is a typical climacteric fruit which has gained a value in human consumption and commercial importance in recent years, attracting researches to study its chemical or nutrient compositions [16,17,18].

Medlar has rich nutritional properties, especially in the mineral content (Al, Ba, Ca, Cu, Co, Fe, K, Li, Mg, Mn, Na, Ni, P, Sr, Ti and Zn), with the highest accumulation of potassium [17]. According to Haciseferogullari *et al.* [19], the potassium content is higher than 8,000 mg·kg^-1^ of fresh weight. Murcia *et al.* [20] also reported that the ripe medlar fruit is an important source of nutritionally important minerals and trace elements, particularly Ca, Cu, Fe, K, Mg, Mn, Na and Zn, for human populations in Southern Europe, Turkey and Iran. Fruits also contain a high content of sugars, organic acids, amino acids and tannins. Only limited papers have been published focused on these phytochemicals, e.g. De Pascual *et al.* [21] analyzed flavanols in medlar fruit. 

In Central Europe, sub-globose or pyriform fruits crowned by foliaceous sepals are harvested in the late autumn [18,22]. Medlars are hard when they are ready to be harvested. They must be allowed to soften and sweeten before becoming edible. The process of softening is called “bletting”. After a frost or cold exposure, fruits on the trees or after harvesting become brown (the pulp darkens) and soft when ready to eat [16,17,19,22]. The harvesting of medlar fruits during the physiological ripening stage and their storaging in straw until over-ripening are well-known traditions, still alive today [17]. Following a harvest period fruits can have a relatively short shelf life during which they undergo profound changes in texture, colour and flavour [23]. Physico-chemical properties of medlar fruits and their remarkable changes during ripening, especially fructose, glucose, sucrose, fatty acids [16], ascorbic acid, and mineral composition were studied by Glew *et al.* [17]. According to Dinner *et al.* [18], chemically, bletting brings about an increase in sugars and a decrease in tannins. Flesh browning is associated with the enzyme polyphenol oxidase (PPO). To our knowledge, there are no data about changes in total antioxidant activity during the development and maturation of medlar fruit. Rodriguez *et al.* highlighted the lack of published data on the physicochemical and chemical changes (sugars, organic acids, minerals, *etc.*) that occur during ripening of medlar [24]. Understanding the biochemical changes in medlar, chemistry of phytochemical transformations in the fruits and their functions in plant physiology, but also in food science, nutrition and health should stimulate an interest in maximizing beneficial sensory and nutritional effects of polyphenols in the diet. 

The aim of this study was to determine the influence of different ripening stages on the content of ascorbic acid, selected mineral elements (phosphorus, potassium, calcium, magnesium, sodium, iron, manganese, zinc, copper, molybdenum) and total phenolic compound content. Furthermore, we aimed at determining the phenolic profile, and evaluating the antioxidant activity of medlar fruits growing in Straznice, the Czech Republic.

## 2. Results and Discussion

Among the anciently cultivated Eurasian fruit crops, many rosaceous trees provide a wide range of edible fruits, which can be classified as neglected or underutilized plants. As mentioned above, a lot of these fruits are known for their great dietary and therapeutic effects, and/or excellent sensory qualities, apart from nutritive and medicinal values. However, several flesh fruits are highly susceptible to enzymatic browning. The medlar meets this criterion, as PPO enzymes associated with flesh browning, which generally results in loss of nutritional, functional and organoleptic qualities, such as darkening, softening and off-flavour development are also active in medlar fruits [25].

### 2.1. Macroelements

Within all determined mineral compounds at ripe stage of medlar fruit, the content of potassium (K) was the highest one (average 8,320 ± 93 mg kg^-1^). This fact is in accordance with the studies of Haciseferogullari *et al.* [19], although we determined a higher level of this element in comparison with this author. Similarly, we found a nine-fold higher accumulation of manganese (Mn), 3.5 times the amount of calcium (Ca) and 2.7 times that of phosphorus (P). Only in case of iron (Fe), we determined a 3.5-fold lower value (in comparison with the studies of Haciseferogullari *et al.* [19]). Consequently, all these differences in mineral composition can be caused by different growth, climatic and soil conditions or a cultivation technique. The content of K in ripe stage of medlar is similar to other kinds of pomaceous fruits, e.g. apples. Moreover, we found a comparatively higher content of dry matter in medlars (from 29.88 to 37.15%). The dry matter concentration was the highest in immature fruits and the lowest in ripe fruits. This fact is in accordance with the observations of Glew *et al.* [17,22,23]. Regarding mineral elements, the content of dry matter was reduced with increasing duration of ripening (Table 1). During the ripening process, we estimated a decrease in the content of some macronutrients, such as K and magnesium (Mg). The highest content of these elements was determined in the first observed ripening stage 134 days after full bloom (DAFB). According to Glew *et al.* [17,22,23], the concentrations of K, Ca, Mg gradually decreased throughout the process of ripening. The maximal level of P and sodium (Na) is obviously determined in the ripe stage of medlar fruit, however, the Mg content varies, which was in good agreement with the results obtained in our study. P reached a similar level at 134 DAFB and 174 DAFB and there were no evidence of statistically significant differences between the ripening stages observed. Similarly, the content of Na fluctuated with the same value of fruit harvested at 144 DAFB and 154 DAFB.

### 2.2. Microelements

All micronutrients maintained the similar value during the ripening process and there were no statistically significant changes. Cold unfavourable weather did not influence the levels of micronutrients (Table 2). In medlar fruit, the levels of iron (Fe) and manganese (Mn) were the highest between the analyzed micronutrients, which is in good agreement with the results obtained in our study. Fe is also a major micronutrient in fruits of lemon, orange and grapefruit [26]. In the micronutrient content, the increasing tendency was observed in the Fe and in the molybdenum (Mo) contents with the similar levels at 144 and 154 DAFB and the same level at 164 DAFB and 174 DAFB, while in the zinc (Zn) content, after decreasing at 144 DAFB we noticed the similar tendency to the increase. The fluctuations of levels were well evident in the Mn content with the similar level at 134 DAFB and 174 DAFB showing the maximum values of this element. The copper (Cu) content increased up to stage 164 DAFB reaching the highest level 5.94 mg kg^-1^ (DM). Although many authors introduce a higher copper content in ripe fruit [27-29], this fact was not proved in our measurement, probably due to low concentrations of Cu in soil (Table 2). On the contrary, the rate between elements and the changes in their content during the ripening stage was in accordance with the papers of Glew *et al.* [17,22,23], Haciseferougullari *et al.* [19], and Romero-Rodriguez *et al.* [24].

Between individual microelements and macroelements we determined mutual correlations, which can prove dependences between monitored elements. The highest values of correlation coefficient were determined for four elements (Ca, Mg, Mo, and Fe). In these elements, we can presume their mutual connection in medlar fruits.

### 2.3. Total phenolic content, total antioxidant activity and ascorbic acid content

The results obtained from total phenolic content assay (Table 4) display a significant decrease of the total phenolic content during the ripening of medlar fruits. At the ripening stage of 134 DAFB, the total phenolic compound content was 170 ± 1 mg gallic acid equivalent (GAE) 100 g^-1^ FM, but at the 174 DAFB stage, *i.e.* after full bloom, the content of phenolics was of 54% of this value (Table 4). However, Parr and Bolwell mentioned [30] that as the fruit ripening progressed through ripe to over-ripe, there was an apparent gradual decrease in the total fruit phenolic concentrations, which is connected with an increased polyphenol oxidase activity. We can assume the certain variability between the total phenolic content in medlar fruits between this study and the previous ones. In connection with the decrease of the phenolic content, the total antioxidant activity also decreased (Table 4). Polyphenols are reducing agents and can react as vitamins C and E and carotenoids with Folin Ciocalteu chemicals [31]. The determination of total phenolic contents by Folin Ciocalteu method is considered unsuitable for the total phenolics determination by many investigators, because the reagent (the mixture of phosphotungstic acid and phosphomolibdic acid) reacts with other non- phenolic reducing compounds and this leads to the overvaluation of the phenolic content. Some of the most interfering compounds in this reaction are organic acids, sugars and amino acids. A way to remove these interferences is using solid phase extraction with C18 columns. The other way is to use high performance liquid chromatography for detection of phenolics but these methods are suitable for determination of specific compounds. 

**Figure 1:**
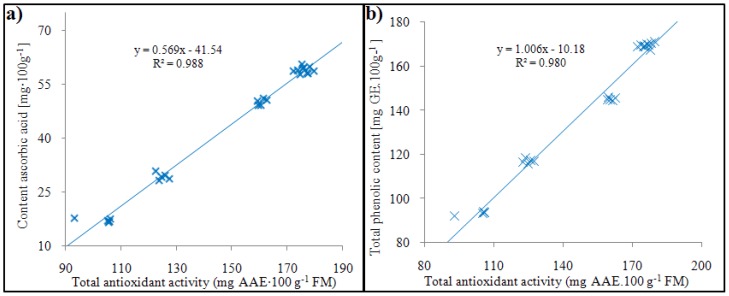
The correlation between the ascorbic acid content and total antioxidant activity (a); the correlation between the total content of phenolic compounds and total antioxidant activity (b).

Several authors have referred to high correlation dependence between low-molecular phenolics and antioxidant activity in fruits [32,33]. The ABTS test used for the detection of antioxidant activity is based on the monitoring of the course of inactivation of the cation ABTS^+^, which is produced during the oxidation of 2,2´-azinobis(3-ethylbenzothiazoline-6-sulphonate). Antioxidant activity measured using the ABTS test in the cultivars of medlar varied after the calculation on ascorbic acid equivalents from 100 to 180 AAE (Table 4). Ascorbic acid (AA, Vitamin C) is a natural inhibitor of PPO [34]. Antioxidants are known to participate in reducing reactions. This fact suggests the possibility of their electrochemical detection. Moreover, the possibility of interference by water soluble UV-absorbing substances, such as proteins, nucleic acids and amino acids should also be considered [35]. The results obtained by liquid chromatography with amperometric detection are listed in Table 4 and shown in Figure 1a. The AA content decreased dramatically through fruit development, which is in accordance with the results of Glew *et al.* [17,22,23]. Notably, AA is known to be prooxidative at low concentrations, whereas it is an important oxygen scavenger at higher concentrations [36]. Several studies have demonstrated a synergistic effect of AA with other antioxidants found in plant material [37]. The differences between individual ripening stages can be considered statistically significant for ascorbic acid, total phenolic content and total antioxidant activity excepting differences between stages 134 DAFB and 144 DAFB. Moreover, the antioxidant activity was correlated with total phenolic content and content of ascorbic acid. It clearly follows for the results obtained that phenolic content and ascorbic acid content are in significant correlation with the ABTS test (Figure 1a,b).

### 2.4. Determination of single phenolic compounds

**Figure 2:**
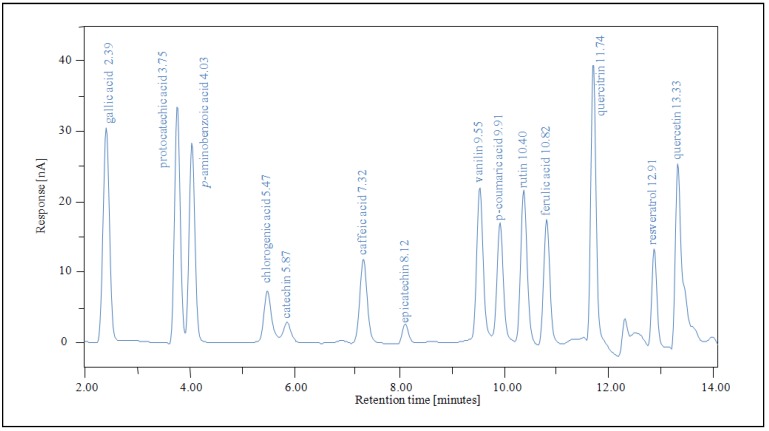
Chromatograms of mixture of standard phenolic compounds (retention times - gallic acid 2.39; protocatechuic acid 3.75; *p*-aminobenzoic acid 4.03; chlorogenic acid 5.47; catechin 5.87; caffeic acid 7.32; epicatechin 8.12; vanillin 9.55; *p*-coumaric acid 9.91; rutin 10.40; ferulic acid 10.82; quercitrin 11.74; resveratrol 12.91; quercetin 13.33).

High performance liquid chromatography with ultra-violet detection (HPLC–UV) represents a very convenient approach for identification and quantification of phenolics in plant extracts [35,38]. Phenolic compounds of edible parts of medlar (*Mespilus germanica* L.), cultivar 'Dutch' were characterized and quantified by HPLC-UV-Vis (Figure 2).

**Figure 3:**
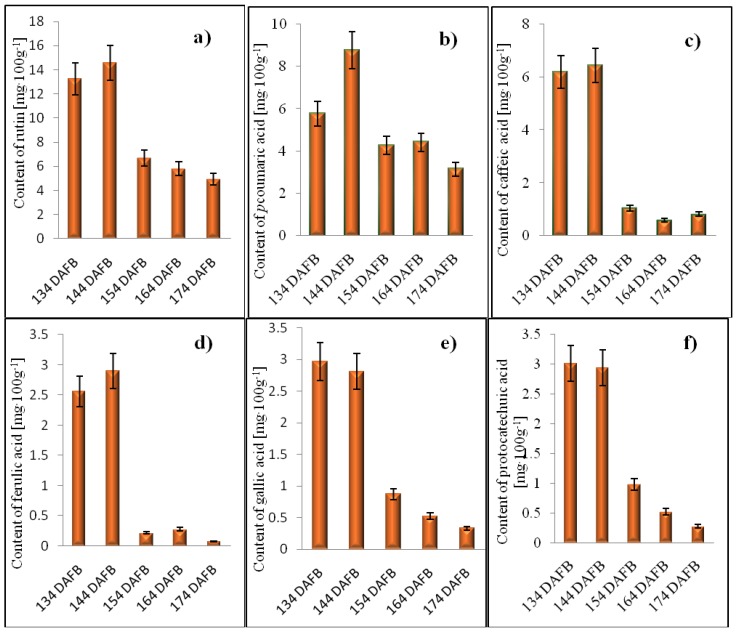

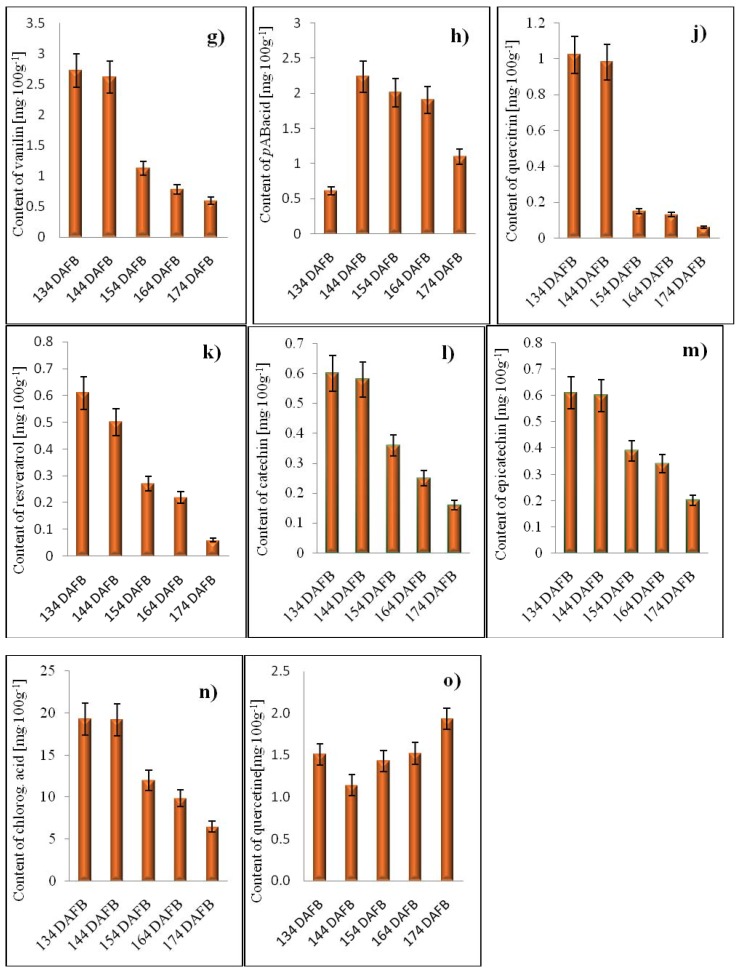
The changes in the content of the following phenolic compounds in the different DAFB: a) rutin, b) *p*-coumaric acid, c) caffeic acid, d) ferulic acid, e) gallic acid, f) protocatechuic acid, g) vanilin, h) *p*-aminobenzoic acid, j) quercitrin, k) resveratrol, l) catechin, m) epicatechin, n) chlorogenic acid. All values are introduced as mg 100g^-1^.

The changes in the content of all detected phenolic compounds are shown in Figure 3. Over the fruit maturation, quercetin and its glycosilated derivates, such as gluco- and rhamnosides, were the most abundant flavonols. Immature fruits contain higher levels of these flavones, which were detected in immature rather than mature fruits [39]. However, the sensory qualities of fruit are extremely complicated. It is difficult to objectively describe the influence of individual compounds. The presence of vanillin can be considered as aroma quality parameter for medlar fruits. One additional flavonoid compound was identified as resveratrol, which predominantly occurs in the epidermis (skin) of wilting berries, a stilbene derivative with strong antioxidant effects *in vivo* [40]. Based on the large set of the results we were interested in the issue whether the content of the individual phenolic compounds correlated with each other according to various ripening stage. Therefore, the statistical treatment of the data obtained was performed at all detected phenolics. The correlation of the certain compounds was calculated according to the following equation:

$$t=\frac{|R|\sqrt{n-2}}{\sqrt{1-R^{2}}}$$

where R is correlation coefficient from 1 to -1, 1 = correlate, 0 = non-correlate, -1 negative correlation, R^2^ is coefficient of determination from 0 to 1, n is number of values = 5, t is significance level

**Figure 4:**
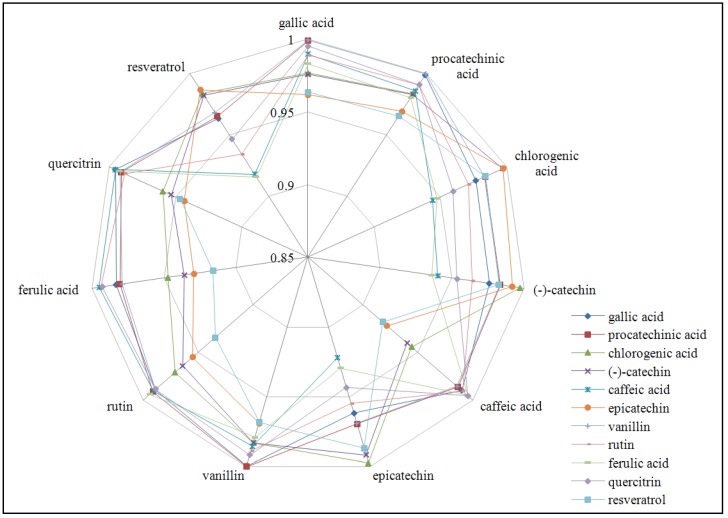
The correlation between the contents of the certain phenolic compounds at P < 0.01.

The results from the statistical analyses of eleven phenolic compounds (gallic acid, protocatechuic acid, chlorogenic acid, catechin, caffeic acid, epicatechin, vanilin, rutin, ferulic acid, quercitrin and resveratrol) are shown in Figure 4. To better present the large amount of the obtained data the undecagon figure was selected. The vertexes of the figure represent single phenolic compounds. The curves demonstrate the correlation coefficient of the certain phenolic compound with those given in the vertex. It clearly follows from the results obtained that all presented compounds correlated with coefficients higher than 0.9 at P < 0.01. The rest of them (*p*-aminobenzoic acid, *p*-coumaric acid and quercetin) did not correlate and therefore are not shown in this figure. The highest correlation among all statistically significant correlation was detected at vanillin.

Although three additional unidentified phenolics were characterized as flavonols, based on UV-VIS spectral analysis, both benzoic acid and cinnamic acid derivates appear to be the major constituents in phenolic fraction of medlar fruits. Hydroxybenzoic acids are found in various fruits and occur mostly as esters, including salicylic acid (2–hydroxybenzoic acid), protocatechuic acid (3.4– dihydroxybenzoic acid), gallic acid (3.4.5–trihydroxybenzoic acid). Hydroxycinnamates, especially neochlorogenic acid and chlorogenic acid, predominated in rosaceous fruits [41]. Seven phenolic acids were identified as gallic, protocatechuic, *p*-aminobenzoic, chlorogenic, caffeic, *p*-coumaric and ferulic acid. The chemoprotective properties of fruits have been partly attributed to phenolics such as gallic and chlorogenic acids. The phenolic content (PC) generally correlates with antioxidant activity for various types of fruits [42].

## 3. Experimental 

### 3.1. Plant material

The full bloom of the medlar source was considered to be on 10 June 2008 and the fruit were sampled at five ripening stages, which were at 134, 144, 154, 164 and 174 days after full bloom (DAFB). At the 134 days stage the skin was green, the pulp white and fruit hard, 144 days after full bloom the skin was light brownish, the pulp white and fruit hard. At the 154 and 164 days stages the skin was getting brown and the pulp was mostly white estimated as consumption maturity when fruits become edible. At the 174 days stage the skin and the pulp were completely brown and soft (this stage leads to the development of the over-ripening stage, where the browning and fruit texture changes occur). The harvested fruits (n = 15) were cleaned, washed in redistilled and deionised water in a mortar. The parts of exocarp and mesocarp were used for the measurement of ascorbic acid content, the total content of phenolic compounds and for total antioxidant activity. For characterization of nutritional value of medlar fruits, above mentioned basic parameters were supplemented with data about the contents of some mineral elements. Each parameter was measured in five replications.

### 3.2. Locality description and collection of samples

**Figure 5:**
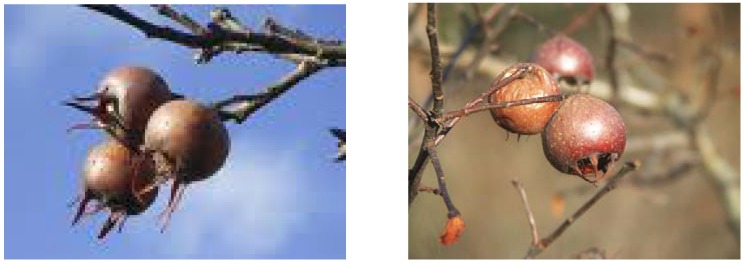
Photos of the medlar fruit (*Mespilus germanica* L.) with the permission from www.ekozahrady.com.

Investigated fruits of medlar (*Mespilus germanica* L., Figure 5), cultivar 'Dutch' were harvested under typical conditions prevalent in the Czech Republic, in the cadastral area of Straznice (17°19′09″V. 48°53′58″S), where the average altitude above sea level is 176 m, the mean annual temperature and precipitation are 8.9 °C and 553 mm, respectively. Soil agrochemical characteristics described in accordance with Kuca *et al.* [43], are presented in Table 5.

Fruits were randomly collected from five trees at every phenological stage. The temperature was measured at 2 meters above ground at 7 a.m. every day. The average values of temperatures and rainfalls during vegetation period from observed area are shown in Table 6. 

Fruits with intact exocarp were transported in liquid nitrogen cooled box to laboratory conditions and kept frozen at -80 °C. Five fruits from each tree were used (*i.e*. altogether 25 per each stage of ripening).

### 3.3. Chemicals

HPLC standards of quercetin, rutin trihydrate and quercitrin dihydrate were obtained from Roth GmbH (Roth GmbH, Karlsruhe, Germany). All other chemicals used were purchased from Sigma Aldrich (Sigma-Aldrich, USA) unless noted otherwise. The stock standard solutions of the reagents were prepared with ACS water (chemicals meeting the specifications of the American Chemical Society, Sigma-Aldrich, USA) and stored in the dark at –20 °C. Working standard solutions were prepared daily by diluting the stock solutions with ACS water. The pH value was measured using inoLab Level 3 (Wissenschaftlich-Technische Werkstatten GmbH; Weilheim, Germany). Deionised water underwent demineralization by reverse osmosis using an Aqua Osmotic 02 (Aqua Osmotic, Tisnov, Czech Republic) instrument and then it was subsequently purified using Millipore RG (Millipore Corp., USA, 18 MΏ) – MiliQ water.

### 3.4. Extraction procedures

The extraction of phenolics was performed according to the method described by Vasantha Rupasinghe *et al.* [44] using the following procedure: 10 g of the fruit matrix were homogenized in an extraction mixture prepared from hydrochloric acid, methanol, ACS water, in the ratio 2:80:18 (v/v). The resulting paste was placed into Erlenmeyer flasks (120 mL) and let to stand in a water bath with the temperature of +50 °C for a period of 2 hours. For the chromatographic determination of the ascorbate content, fruits were homogenized in ACS water. Thereafter, the suspension obtained was centrifuged for 10 minutes (at 16,000 rpm). Subsequently, the supernatant was filtered under vacuum through the glass frit. A volume of 30 mL of the extract was vacuum evaporated to a final volume of approximately 5 mL. The total solids were quantitatively transferred into Eppendorf beakers (10 mL) and diluted up to 10 mL with ACS water.

### 3.5. Determination of total phenolic content 

For the measurement of total contents of phenolic substances, the extract (0.5 mL) was sampled and diluted with water in a 50-mL volumetric flask. Thereafter, Folin-Ciocalteau reagent (2.5 mL) and a 20-percent solution of sodium carbonate (7.5 mL) were added. The resulting absorbance was measured in the spectrophotometer LIBRA S6 (Biochrom Ltd., Cambridge, UK) at the wavelength of 765 nm against a blank, which was used as reference. The results were expressed as mg of gallic acid 100 g^-1^ of fresh matter (FM).

### 3.6. Antioxidant activity assay 

Antioxidant activity was measured using the method described by Sulc *et al.* [45]. This test is based on monitoring the course of inactivation of the cation ABTS^+^, which is produced during the oxidation of 2,2´-azinobis (3-ethylbenzothiazoline-6-sulphonate). ABTS^+^ shows a strong absorbance in the visible region of the electromagnetic spectrum (600–750 nm); this solution is green and its antioxidant activity can be easily measured by means of spectrometry. A quantity of ABTS (54.9 mg) was dissolved in phosphate buffer (20 mL, pH 7.0; 5 mM) and activated on cation radical of ABTS^+^ by means of addition of MnO^2+^ (1 g). The resulting solution was intermittently stirred for an activation period of 30 min. Thereafter, the solution was centrifuged for 5 min. at 7,000 rpm and filtered through a syringe filter (0.25 µm). A volume of the filtrate (2 mL) was diluted with phosphate buffer to the absorbance (t_0_) of 0.500 ± 0.01, which was measured at the wavelength of 734 nm. After measuring the absorbance at time t_0_, the sample (0.5 mL) was added and the new absorbance value was measured at time t_20_, *i.e.* after 20 minutes. Antioxidant activity was calculated as a decrease in the absorbance value using the formula: (%) = 100 − [(At_20_/At_0_) × 100]. The results of absorbance were converted using a calibration curve of the standard and expressed in ascorbic acid equivalents (AAE).

### 3.7. Mineral content assay 

The samples were dried to a constant weight in a drier at 105 °C ± 2 °C. Thereafter, homogenized dry matter (1 g, with a particle size of 1 mm) was subsequently mineralised in a mixture of concentrated sulphuric acid with 30 % addition of hydrogen peroxide. After the mineralization, the obtained samples were quantitatively transferred into a 250 mL volumetric flask and filled to the volume with re-distilled water. The resulting homogenate was determined using atomic absorption spectrometer PHILIPS PU 9200X (Germany). The results obtained were expressed as mg kg^-1^ of dry matter (DM).

### 3.8. Determination of ascorbic acid

The determination of the ascorbic acid content was carried out by optimized method of Gazdik *et al.* [46]. The sample (5 g) was homogenized using a mortar by adding acetonitrile (25 mL) and 0.09% trifluoroacetic acid in the ratio 3:97 (v/v). The extracts obtained were 100 × diluted with ACS water and transferred into a volumetric flask and diluted with ACS water. The flask with the samples was placed into a water bath with the temperature of 25 °C where the samples were extracted for 15 min, filtered through 0.45 μm Teflon membrane filter prior to the measurement. To keep out the samples of daylight, the flask was covered with aluminium foil during the preparation. The measurements of the samples were carried out immediately after the preparation steps. The chromatographic conditions were as follows: temperature: 30 °C, mobile phase – acetonitrile and 0.09% trifluoroacetic acid in a 3:97 (v/v) ratio. The HPLC-ED system consisted of a solvent delivery pump operating in the range of 0.001−9.999 mL min^-1^ (Model 583 ESA Inc., Chelmsford, MA, USA), a guard cell (Model 5020 ESA, USA), chromatographic column – MetaChem Polaris C18A 150 × 2.0 mm, 3 µm particle size) and an electrochemical detector (ED) that included low volume flow-through analytical cells (Model 5040, ESA, USA), which consist of a glassy carbon working electrode, palladium electrode as reference electrode and an auxiliary carbon electrode, and Coulochem III as a control module. The glassy carbon electrode was polished mechanically with 0.1 μm alumina (ESA Inc., USA) and sonicated at laboratory temperature for 5 min using a Sonorex Digital 10 P Sonicator (Bandelin, Berlin, Germany) at 40 W. The sample (5 μL) was injected automatically. The data obtained were treated by CSW 32 software. The experiments were carried out at room temperature. The content of ascorbic acid was calculated on a mg 100 g^-1^ fresh matter basis.

### 3.9. Determination of phenolic compounds

The HPLC-UV-Vis system consisted of two solvent delivery pumps operating within the range of 0.001−9.999 mL min^-1^ (Model 582 ESA Inc., Chelmsford, MA), Metachem Polaris C18A reverse-phase chromatographic column Zorbax SB C18 (150 × 4.6; 5 µm particle size, Agilent Technologies, USA) and UV detector Shimadzu (Model 528, ESA, USA). Both the detector and the column were thermostated. The sample (15 μL) was injected using an autosampler (Model 540 Microtiter HPLC, ESA, USA). The chromatographic conditions were optimized –mobile phase flow 1 mL min^-1^, temperature 30 °C. Isocratic mobile phase was as follows: A: acetic acid (50 mM) and B: acetic acid (50 mM) in acetonitrile. The UV detector was scanned at 260 nm [47].

### 3.10. Statistical analysis

The data obtained were statistically analysed using analysis of variance (ANOVA) and Tukey’s multiple range test for comparison of means using the statistical package Unistat, v. 5.1. 

## 4. Conclusion

Ripe medlar fruit is an important source of minerals and trace elements, in particular K, Ca, Mg, P, Cu, Fe, Mn, Mo and Na. Our findings may also be useful for dietary information and determine changes in the total phenolic content, antioxidant activity and the mineral (elements) content during medlar fruit ripening. Furthermore, the results of our study demonstrate the fact that monitored ascorbic acid, total polyphenolics and total antioxidant activity were variable and display a decreasing tendency, thus, special attention should be given to utilizing unripe medlar fruit for food processing, *etc.* On the other hand, fruits become edible only after natural softening and browning (ripe stage of fruits). A decreasing tendency in potassium, calcium and magnesium contents throughout the studied ripening stages was also found. Contrariwise, the contents of all micronutrients, phosphorus and sodium were balanced, with no statistically significant differences between the monitored ripening stages. It can be considered as a positive fact for ideal consumption quality of fruits.

## Figures and Tables

**Table 1:** The changes in the content of macroelements of phosphorus, potassium, calcium, magnesium and sodium during 5 different ripening stages. All values are introduced as mg kg^-1^ (dry matter, DM).

	Ripening stage (DAFB)				
**Element**	**134**	**144**	**154**	**164**	**174**
**Phosphorus**	939 ± 46	945 ± 35	940 ± 43	961 ± 41	938 ± 32
**Potassium**	8766 ± 101	8751 ± 85	8737 ± 77	8725 ± 92	8320 ± 93
**Calcium**	3094 ± 82	3111 ± 79	3095 ± 88	2754 ± 86	2695 ± 115
**Magnesium**	1038 ± 40	1035 ± 44	1021 ± 31	913 ± 50	842 ± 41
**Sodium**	118 ± 19	115 ± 17	115 ± 15	124 ± 12	121 ± 16

**Table 2:** The changes in the content of microelements of iron, manganese, zinc, copper and molybdenum during five different ripening stages. All values are introduced as mg kg^-1^ (dry matter, DM).

	Ripening Stage (DAFB)				
Element	134	144	154	164	174
Iron	27.05 ± 2.04	27.14 ± 1.25	27.35 ± 1.67	27.52 ± 2.20	27.60 ± 1.45
Manganese	14.99 ± 2.11	14.25 ± 2.57	14.52 ± 2.50	14.17 ± 2.96	14.95 ± 2.15
Zinc	5.82 ± 0.51	5.71 ± 0.44	5.80 ± 0.67	5.90 ± 0.39	6.10 ± 0.50
Copper	5.37 ± 0.92	5.43 ±1.10	5.66 ± 0.79	5.94 ± 0.86	5.10 ± 1.13
Molybdenum	0.50 ± 0.05	0.50 ± 0.05	0.53 ± 0.05	0.60 ± 0.05	0.60 ± 0.05

**Table 3:** Mutual correlation of four elements found in medlar fruit extracts. The values of correlation coefficients are of P < 0.01.

	Calcium	Magnesium	Molybdenum	Iron
**Calcium**	X	0.980	X	X
**Magnesium**	0.980	X	-0.969	X
**Molybdenum**	X	-0.969	X	0.960
**Iron**	X	X	0.960	X

**Table 4:** The total phenolic content, total antioxidant activity and the ascorbic acid content of extracts of fruits.

Days after full bloom	Ascorbic acid content (mg·100 g^-1^ FM)	Total phenolic content (mg GAE·100 g^-1^ FM)	Total antioxidant activity (mg AAE·100 g^-1^ FM)
134	59 ± 2	170 ± 1	180 ± 4
144	58 ± 2	169 ± 1	175 ± 5
154	50 ± 2	145 ± 1	160 ± 4
164	29 ± 2	117 ± 1	120 ± 5
174	17 ± 1	93 ± 1	100 ± 4

**Table 5:** Soil agrochemical characteristics. All values are introduced as mg kg^-1^.

pH	P	K	Ca	Mg	Mn	Cu	Zn	Mo
6.8	33	122	2411	186	514	4.5	17.4	2.5

**Table 6:** Climatic characteristics.

Month	Temperature [°C]	Precipitation [mm]	Atmospheric moisture [%]	Global radiation [w · m^-2^]
March	3.8	25	0.7	75
April	9	49	47.0	189
May	13.7	57	54.0	198
June	20.3	47	59.9	210
July	19.9	71	59.0	193
August	14.3	38	66.5	175
September	10.3	29	80.7	117
October	6.7	13	80.1	65
November	2.2	29	82.9	36
